# Supplementary material for: The causal relationship between the human gut microbiota and pyogenic arthritis: a Mendelian randomization study
Source: Front Cell Infect Microbiol. 2024 Nov 26;14:1452480. doi: 10.3389/fcimb.2024.1452480 (PMC11629706; doi:10.3389/fcimb.2024.1452480)
Supplement: Supplementary file 1 [file DataSheet1.zip › mendelian test/ebi-a-GCST90027743.csv_leave_one_out.pdf]

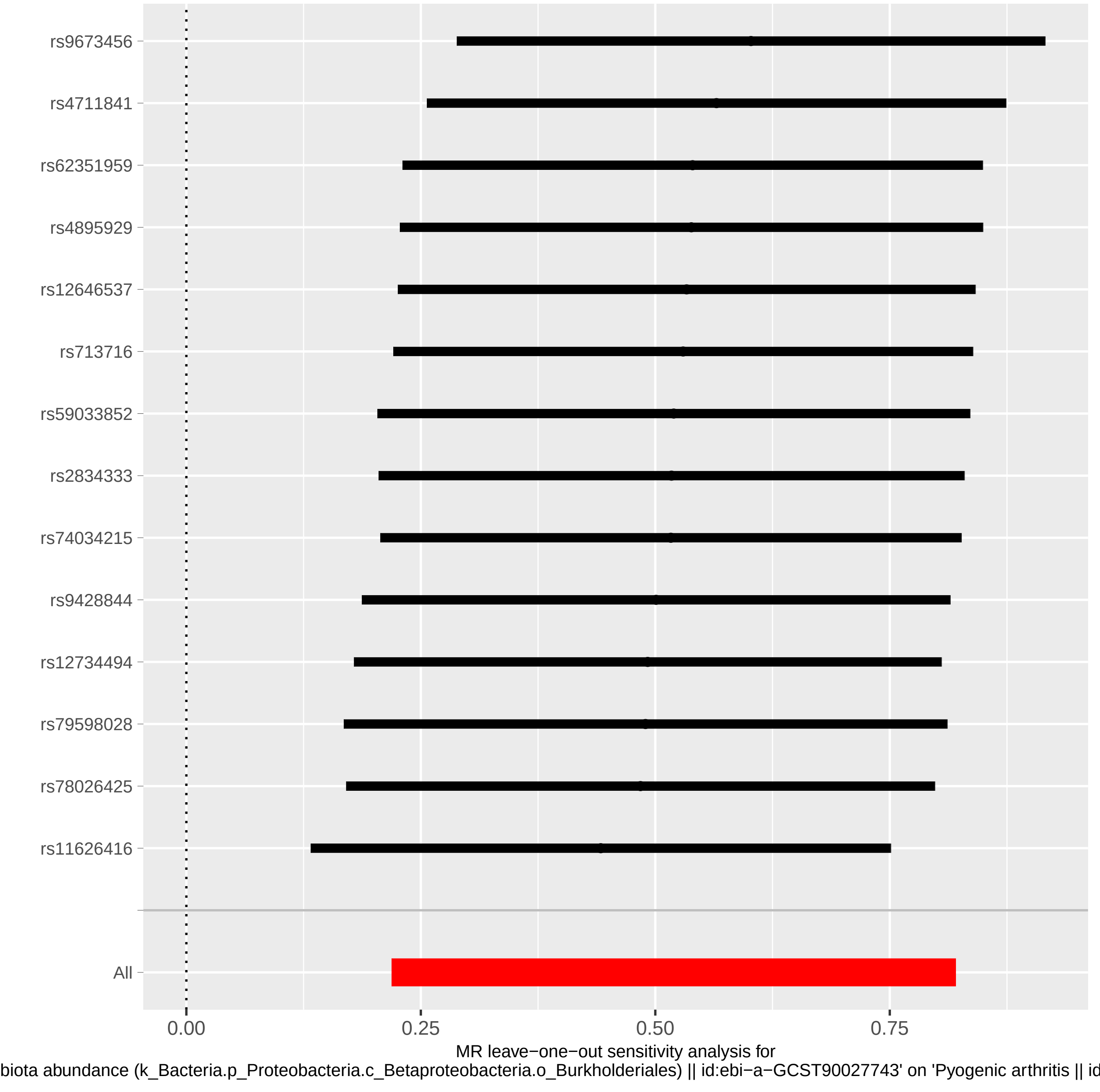
rs9673456
rs4711841
rs62351959
rs4895929
rs12646537
rs713716
rs59033852
rs2834333
rs74034215
rs9428844
rs12734494
rs79598028
rs78026425
rs11626416
All
0.00
0.25
0.50
0.75
MR leave−one−out sensitivity analysis for
biota abundance (k_Bacteria.p_Proteobacteria.c_Betaproteobacteria.o_Burkholderiales) || id:ebi−a−GCST90027743' on 'Pyogenic arthritis || id
